# Impaired Emotional Self-Referential Processing in First-Episode Schizophrenia

**DOI:** 10.3389/fpsyt.2021.591401

**Published:** 2021-04-07

**Authors:** Yanli Zhao, Zhiren Wang, Yueyao Zhang, Yuanyuan Zhang, Jinguo Zhang, Dong Li, Chunling Xiao, Shuping Tan, Dandan Zhang

**Affiliations:** ^1^Psychiatry Research Center, Beijing Huilongguan Hospital, Peking University Huilonguan Clinical Medical School, Beijing, China; ^2^College of Psychology, Shenzhen University, Shenzhen, China; ^3^School of Psychology, North China University of Science and Technology, Tangshan, China

**Keywords:** self-reference effect, first-episode schizophrenia, self-referential processing, mother-referential processing, negative bias

## Abstract

Impairments in self-representation are relevant to the expression of psychosis. To date, the characteristics and neural mechanisms of self-impairment in schizophrenia remain unclear. To this end, we used event-related potentials (ERPs) to measure brain activity in 56 first-episode patients with schizophrenia and 56 healthy controls. Participants judged personal trait adjectives regarding themselves, their mothers, or a public person, followed by an unexpected old/new recognition test. The recognition score for mother-reference adjectives was lower than that for self-reference adjectives in patients, while the control group showed comparatively high recognition scores for both self- and mother-referential adjectives. In addition, control subjects recognized more negative words, while patients remembered more positive words. ERP data revealed that controls exhibited typical task effects (self-reference = mother-reference > other-reference) during both automatic attention and effortful encoding periods [indexed by P2 and the late positive potential (LPP), respectively]. In contrast, patients only exhibited the task effect in the P2 amplitude. Moreover, controls exhibited larger P2 amplitudes during encoding negative than positive words, whereas patients had enhanced LPP amplitudes during memory retrieval of positive compared to negative words. These findings demonstrated self-representation dysfunction in first-episode schizophrenic patients in mother (the intimate other) referential processing and the absence of a negative memory bias.

## Introduction

There is a long-standing tradition of understanding core psychotic symptoms as ego-disturbances (1). The term ego contains two important concepts of self. The first is “the minimal self (pre-reflective self),” which enables us to perceive ourselves as the immediate subject of experience. The other one is the “narrative self (reflective self),” which comprises all aspects of our personality and allows us to understand ourselves in the continuity of time (2, 3). It has been found that some patients with schizophrenia who suffer from symptoms such as thought insertion also have disrupted sense of self-agency, which is one core aspect of minimal self-awareness (3). Moreover, a disturbance of reflective self may cause changed concept of self and meta-cognition of others (4). The present study intends to focus on the aspect of reflective self in schizophrenia.

The most commonly used paradigm to address reflective self in neuro-scientific research is self-referential processing or self-reference effect (SRE) task. Self-referential processing refers to a conscious process in which a decision is made regarding oneself and accurately represents one's traits, abilities and attitudes (5). Impaired self-referential processing is relevant to maladaptive social functioning and poor illness awareness of schizophrenia (6–8). Moreover, some brain imaging studies have investigated the neural substrates underlying the self-referential processing in schizophrenia and found abnormalities in cortical midline structures (9–11). Additionally, one functional magnetic resonance imaging (fMRI) study addressed the abnormal neural correlates of self-referential information recognition in schizophrenia, revealing that controls showed differential activation within the medial prefrontal cortex (mPFC), ventrolateral prefrontal cortex, temporo-parietal junction, and the posterior cingulate cortex between self and other conditions, while patients did not (12). Prior research in schizophrenia (13, 14) also assessed event-related potentials (ERPs), which have excellent temporal resolution in the millisecond range to uncover the impaired temporal processes associated with self-referential processing. These studies found that schizophrenia was characterized by reduced P2 (13, 14) and late positive potential (LPP) amplitudes (13) in self-referential conditions over frontal-central cortical regions. The frontal P2 component reflects the automatic monitoring of information (15, 16); and the LPP reflects the sustained engagement and effort allocation during strategic cognitive processing (17). Therefore, these ERP findings suggested that the deficits of self-referential processing in schizophrenic patients occurs in both automatic attention and strategic cognitive processing. In addition, the N400 component can also reflect the disturbed self-referential processing in schizophrenia. For instance, Metzler et al. (4) found that increased negative N400 was associated with trait adjectives incongruent compared to congruent with self-concept in normal controls, while this effect was diminished in schizophrenia (4).

SRE task is a common behavioral paradigm to explore self-referential processing. In a typical SRE task, participants are first asked to judge whether a personality trait word is suitable to describe the self or others. After the encoding phase, subjects are required to finish an unexpected old/new recognition test (i.e., the retrieval phase). Substantial evidence has demonstrated that information is better remembered when it is processed in reference to the self than to others in healthy adults (called as SRE) (18). However, an abolished SRE was revealed in schizophrenia (13, 19), providing behavioral evidence of the impaired self-referential processing in schizophrenia. In our opinion, the following aspects need to be further concerned to understand the self-referential deficits in schizophrenia comprehensively.

First, disturbances in self-referential processing have been proposed to represent a potential psycho-pathological trait marker of schizophrenia-spectrum disorders (20, 21). However, most previous studies have found impaired self-referential processing in patients with chronic schizophrenia (10, 11, 13, 14), leaving anti-psychotics-naïve patients and patients with first-episode schizophrenia untested. The investigation of first-episode schizophrenic patients in this study can minimize the confounding effect of long-term medication use, and helps to answer whether the impairment of self-referential processing is the trait maker of schizophrenia (i.e., present in both early and chronic courses of the illness).

Second, studies also found that there is biased processing of emotional self-referent information in healthy adults, although the results remain inconsistent. Some studies found that positive information is better recalled than negative information when it is processed in reference to the self, but not when it is processed in reference to another person (22, 23). However, other studies revealed that negative personality traits are better recognized than positive ones, no matter whether they are processed in reference to the self or another person (22, 24). D'Argembeau et al. (22) proposed that this inconsistency can be influenced by retrieval conditions (22). While a positive bias (positive adjectives are better recalled) in the self-referent condition was often observed in a recall task (22, 23, 25, 26), a negative bias can be found in the recognition task (22). Furthermore, in line with the aforementioned behavioral results, the P2 and LPP amplitudes have been observed to be larger for positive compared with negative self-referential words (26, 27), and for negative compared with positive ones in healthy subjects (28, 29). Some studies focus on the emotional self-referential processing biases in depression (26, 27), but few studies have investigated this emotional bias in schizophrenia.

To address the above gaps in the literature, the present study used the ERP technique to examine: [1] whether first-episode schizophrenic patients would demonstrate deficits in self-referential processing and SRE; [2] whether there are biased processing of emotional self-referent information in first-episode schizophrenia; and [3] whether the neural correlates (ERP indexes) of self-referential processing differ between first-episode schizophrenia and controls during the encoding and retrieval phases of the task. According to previous studies in non-first episode schizophrenia (6, 10, 11, 13, 19), we expected that first-episode schizophrenia may also have self-referential processing impairment and abolished SRE, or while their core self-function is preserved, their relational self may be impaired as they are in the early course of the illness. In the present study, recognition rather than recall task was used (22), thus we hypothesized that a negative bias can be found in normal controls. However, as schizophrenic patients may display distorted attributions of positive and negative traits to self and others so to maintain self-esteem (30), they may show different emotional processing bias from controls. Finally, we expected that healthy controls would show task effect (self = mother > other) and negative bias indexed by the P2 and LPP amplitudes (31), but these effects would be changed in patients, since previous studies (6, 10–12) have observed dysfunction during both encoding and retrieval stages of the SRE task in schizophrenia.

## Methods

### Subjects

The subjects came from the Schizophrenia Spectrum Disorder Project of Beijing Huilongguan Hospital. All patients were inpatients of Beijing Huilongguan Hospital and normal controls were recruited from the surrounding community and university. The inclusion criteria of schizophrenia were: [1] comply with the diagnostic criteria for schizophrenia disorders in the fourth edition of the Statistical Manual of the Schizophrenia (32); [2] aged from 16 to 60 years old; [3] received more than 9 years of education; [4] first episode of illness defined either as first treatment contact or duration of illness up to 3 years following illness onset; [5] antipsychotic naïve or minimal exposure (≤2 weeks antipsychotic treatment); [6] Positive and Negative Syndrome Scale (PANSS) (33) total score ≥ 60, or PANSS positive symptoms with at least one of P1, P2, P3, P6 ≥ 4; [7] volunteered to participate in the study and signed an informed consent form. The exclusion criteria for patients were: [1] Intellectual disability (IQ < 70 based on medical records) or brain organic disease; [2] severe depression, anxiety or substance abuse or dependence in the past 6 months; [3] impairments in hearing or visual perception; [4] serious physical illness or side effects; [5] ongoing medication with immunomodulatory, neurotrophic agents, antioxidants in the past 8 weeks; [6] severe recession or impulsiveness; [7] electroconvulsive therapy treatment in the past 6 months or transcranial magnetic stimulation in the past 2 months. The inclusion criteria for healthy controls were: [1] screened with SCID-I/NP (34) and SCID-II (35) to guarantee that they did not have schizophrenia, schizophrenic affective disorder, depression, anxiety disorder, bipolar affective disorder, or other mental disorders; [2] no family history of mental illness; [3] age and education level requirements consistent with patients; [4] volunteered to participate in the study and signed an informed consent form. The exclusion criteria are the same as the first five exclusion criteria for patients. The experimental protocol was approved by Beijing Huilongguan Hospital.

Sample size calculation was performed with the G^*^Power 3.0.10 software, with 0.812 as the effect size, 5% as the significance level, and 0.90 as the power value. This yielded 33 individuals per group. In fact, this study recruited 61 first-episode schizophrenia subjects and 58 normal subjects. The data of 7 subjects were removed because of a poor EEG signal-to-noise ratio. Eventually, 56 first-episode patients with schizophrenia and 56 normal controls were included in the data analysis. There were no significant differences between the two groups with respect to age, education or gender (Table 1).

**Table 1:** Demographic and clinical data for first-episode schizophrenic patient and the control groups.

**Characteristics**	**Patient** ** (*n* = 56)**	**Control** ** (*n* = 56)**	**Statistics**
Mean age (years)	28.38 (15–47)	29.07 (16–51)	*F*_1, 111_ < 1
Education time (years)	14.29 (9–19)	15.02 (9–19)	*F*_1, 111_ = 2.037, *p* = 0.156
Sex, male/female	24/32	26/30	$\chi_{1}^{2}$ = 0.145, *p* = 0.849
Handedness, right/left	56/0	56/0	
Duration of illness (months)	13.1 (0.5–38)		
Age at disease onset (years)	27.61 (14–47)		
PANSS total score	75.90 ± 10.41		
Positive score	21.64 ± 4.65		
Negative score	17.95 ± 5.40		
General score	36.30 ± 6.28		
Neuroleptic, typical/atypical/both/not taking any medication^a^	1/40/8/4		
Chlorpromazine equivalents (mg/day, 40)	347.8 ± 168.4		

*Descriptive data are presented as mean (range) or mean ± standard deviation*.

a *The medication data were missed for three patients*.

### SRE Task

**Figure 1:**
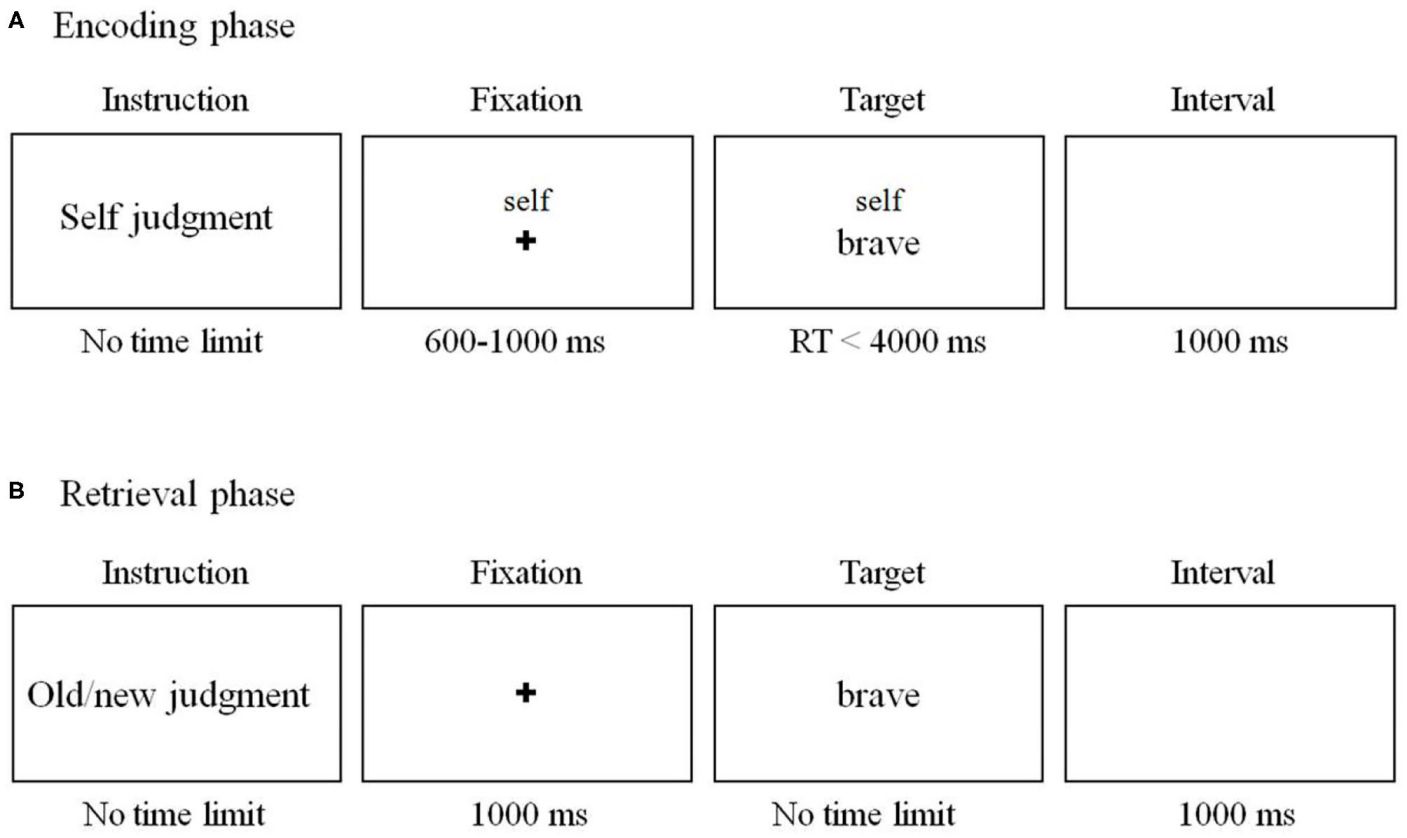
Schema of the self-referential effect (SRE) task in the current study. **(A)** Encoding phase. The stimuli and procedure of mother- and other-referential conditions were the same as those of the self-referential condition except that the word “self” on the screen was replaced by “mother” and “Jintao Hu.” **(B)** Sample stimuli and schematic of a sample trial in retrieval phase.

We used the SRE task as in previous research (36) with slight modifications (Figure 1). The task includes two phases (encoding phase and recognition phase). During the encoding phase (Figure 1A), subjects were required to judge whether an adjective was proper to describe the self, mother, and other (the former chairman of the People's Republic of China, Jintao Hu).

A total of 300 personality-trait adjectives (150 positive and 150 negative adjectives) were selected from established personality trait adjective pools (37). Among them, 180 words were randomly assigned to the encoding phase, while the remaining 120 words were used in the recognition phase. There were 60 words (30 positive and 30 negative words) in each condition during encoding phase. Each trial began with a 600–1,000 ms fixation cross, followed by a “cue” word (either self, mother or other) above the trait adjective during which the subject indicated their response. The word disappeared until the subjects made a choice. Participants were asked to watch an irrelevant movie for 20 min after the encoding phase. Then, they were required to complete an unexpected old/new recognition test (Figure 1B). All the 180 words were included together with another 120 new trait adjective words. Words were presented in a random order. Participants were required to judge whether the word was presented in the encoding phase. There was no time limit for the response. The word disappeared until the subjects made a choice.

### Behavioral Measures

Stimulus display and behavioral data acquisition were conducted using E-Prime software (Version 2.0, Psychology Software Tools, Inc., Pittsburgh, PA). For the encoding phase, self-, mother- and other-endorsement was calculated separately for positive and negative words as the percentage of words the participants endorsed as self, mother-, or other-description. Moreover, we analyzed the reaction time (RT) of the endorsement under different conditions. For the recognition phase, we used recognition score to measure the recognition performance during the old/new memory test. The recognition score was defined as the proportion of hits minus the proportion of false alarm in each condition (38–40). To obtain a comprehensive measurement of the SRE and self-representation, we defined the SRE bias score as the differential recognition score between self- and other-reflection conditions and defined the mother-referential effect (MRE) bias score as the differential recognition score between mother-and other-reflection conditions.

### EEG Recording and Analyses

Brain electrical activity was recorded during both encoding and retrieval phases by a 64-channel amplifier (Brain Products, Gilching, Germany). Online reference electrode was placed at left mastoid. Besides referential and electrooculogram electrodes, a 59-channel EEG data was collected with electrode impedances kept below 5 kΩ. EEG signals were continuously sampled at 1,000 Hz and filtered within 0.01–100 Hz.

Data analyses were performed using Matlab R2011a (MathWorks, Natick, USA) and SPSS Statistics 20.0 (IBM, Somers, USA). The recorded EEG data was down-sampled to 250 Hz and band-pass filtered with a 0.01–30 Hz filter. Then data were referenced to averaged mastoids. EEG segments containing large line noises and myoelectricity were manually rejected. Ocular artifacts were removed from the EEG using a regression procedure implemented in the commercial software Scan 4.3 (Compumedics Neuroscan, El Paso, TX, USA). Cleared data were segmented in association with experimental conditions: beginning 200 ms prior to the word presentation and lasting for 1,700 ms. Epochs were baseline corrected with respect to the mean voltage over the 200 ms preceding word presentation.

According to the ERP topographies, the grand waveform and the relevant literature (17, 18, 22, 23, 31), the present study focused on the frontal P2 and LPP during the encoding phase and focused on the parietal LPP during the retrieval phase. The amplitudes of components were calculated using the mean amplitude within a time window when components reached their peak. In particular, during the encoding phase, P2 was measured at AF3, AF4, F3, and F4 during the time window of 200–300 ms; and the LPP was measured at AF3, AF4, F3, and F4 during the time window of 800–1,200 ms. During the retrieval phase, the LPP was measured at Pz, CPz, P1, and P2 during the 600–1,400-ms time window. In addition to mean amplitude, this study also examined the peak latency of ERP components. Peak latency was manually detected in individually averaged waveforms in each condition.

### Statistics

For behavioral data in the encoding phase, the distributions of endorsed positive and negative self-reference words, positive mother-reference words, and positive and negative other-related words did not obey normal distribution in the normal subjects (Kolmogorov-Smirnov test, all *p* < 0.050). In addition, the distributions of endorsed positive and negative other-reference words did not obey normal distribution in patients (Kolmogorov-Smirnov test, both *p* < 0.01). Therefore, non-parametric tests were used to assess group differences. We used a Wilcoxon signed-rank test to assess whether there was a difference between positive and negative endorsement for each group and used a Mann-Whitney-Wilcoxon rank-sum test to assess whether there was a difference in endorsed positive and negative words between the two groups. Except for the RT under the conditions of self-positive, mother-positive and mother-negative in the normal group, the other conditions were all with a non-normal distribution of RT data. Therefore, the RT data was also analyzed using non-parametric test.

For behavioral data in the recognition phase, all the recognition scores were normally distributed. Therefore, a repeated measures ANOVA was conducted for the recognition score, with two within-subject factors, i.e., task (self/mother/other) and emotion (positive/negative), and one between-subject factor, group (patient/control). Independent-samples *t*-tests were conducted for the comparison between groups on the SRE/MRE bias score.

For ERP data, a repeated measures ANOVA was conducted for the mean amplitudes, with two within-subject factors, task and emotion, and one between-subject factor, group. *P*-values were corrected using the Greenhouse–Geisser correction. Significant interactions were analyzed using a simple effects model and multiple comparisons were adjusted using the Bonferroni test. In addition, a two-tailed Pearson correlation analysis was performed between behavioral data, ERP measures, and clinical data (i.e., PANSS score).

## Results

### Behavioral Results

#### Encoding Phase

##### Word Endorsement

Compared with the patients, healthy controls endorsed more positive words across the three conditions (self: *z* = −4.01, *p* < 0.001; mother: *z* = −3.50, *p* < 0.001; other: *z* = −1.96, *p* = 0.050) and fewer negative words in both self-and mother-related conditions (*z* = −4.70, *p* < 0.001; *z* = −3.17, *p* = 0.002; *z* = −0.39, *p* = 0.699). Moreover, both groups endorsed more positive words than negative ones across the three conditions (all *z* ≤ −5.98, *p* < 0.001). See Table 2.

**Table 2:** Encoding endorsed data for healthy controls and first-episode schizophrenia.

**Task**	**Control** ** (*n* = 56)**	**Patient** ** (*n* = 56)**
**Endorsed (%)**	**Median** ** (minimum, maximum)**	**Median** ** (minimum, maximum)**
Self _positive	93.33 (57, 100)	80.00 (7, 100)
Self _negative	10.00 (0, 40)	26.67 (0, 90)
Other _positive	25.00 (3, 53)	35.00 (10, 73)
Other_ negative	3.33 (0, 40)	3.33 (0, 83)
Mother _positive	95.00 (63, 100)	88.33 (27, 100)
Mother_negative	25.00 (3, 53)	35.00 (10, 73)

##### Response Time

Relative to normal subjects, patients responded slower for positive words (*z* = 4.20, *p* < 0.001) but faster for negative words (*z* = −2.61, *p* = 0.009) in self-relevant condition. Moreover, compared to healthy controls, they responded slower with both positive (*z* = 4.04, *p* < 0.001) and negative (*z* = 2.71, *p* = 0.007) words in mother-relevant conditions. There was no significant difference for either positive (*z* = 0.17, *p* = 0.865) or negative (*z* = −0.07, *p* = 0.947) words in other-relevant condition. In the self-relevant condition, the identification time of patients for negative words was shorter than that for positive words (*z* = −4.93, *p* < 0.001), while there was no significant difference between the identification time for negative and positive words in healthy controls (*z* = −1.14, *p* = 0.253). In the other-relevant condition, both groups recognized the negative words with shorter time than the positive ones (normal: *z* = −2.96, *p* = 0.003; patient: *z* = −2.18, *p* = 0.030). However, in the mother-relevant condition, both groups recognized the negative words for longer time than the positive ones (normal: *z* = 5.02, *p* < 0.001; patients: *z* = 5.45, *p* < 0.001). Moreover, for normal subjects, the identification time for positive mother-words was shorter than that for positive self-words (*z* = −2.25, *p* = 0.025), while there was no significant difference between positive other- and self-words (*z* = −1.38, *p* = 0.169), or between positive other- and mother-words (*z* = −0.12, *p* = 0.902). For patients, the identification times for both positive self- (*z* = 3.88, *p* < 0.001) and mother-words were longer than that for positive other-words (*z* = 3.30, *p* = 0.001), while there was no significant difference between the two former conditions (*z* = −1.08, *p* = 0.279). Both groups recognized the negative mother-words for a longer time than the negative self- (*z* = 3.35, *p* = 0.001; *z* = 5.21, *p* < 0.001, for normal and patient) and other- ones (*z* = −4.94, *p* < 0.001; *z* = −5.18, *p* < 0.001, for normal and patient). There was no significant difference between negative self-and other-ones (*z* = −1.79, *p* = 0.074; *z* = 1.22, *p* = 0.221, for normal and patient). See Table 3.

**Table 3:** Encoding RT data for healthy controls and first-episode schizophrenia patients.

**Task**	**Control** ** (*n* = 42)**	**Patient** ** (*n* = 42)**
**RT (ms)**	**Median** ** (minimum, maximum)**	**Median** ** (minimum, maximum)**
Self _positive	733 (491, 1,078)	912 (505, 1,918)
Self _negative	686 (105, 994)	166 (100, 982)
Other _positive	729 (113, 908)	741 (102, 999)
Other_ negative	554 (100, 990)	510 (100, 995)
Mother _positive	726 (474, 1,024)	882 (540, 1,932)
Mother _negative	910 (396, 1,424)	1,016 (581, 1,938)

#### Recognition Phase

##### Recognition Score

**Figure 2:**
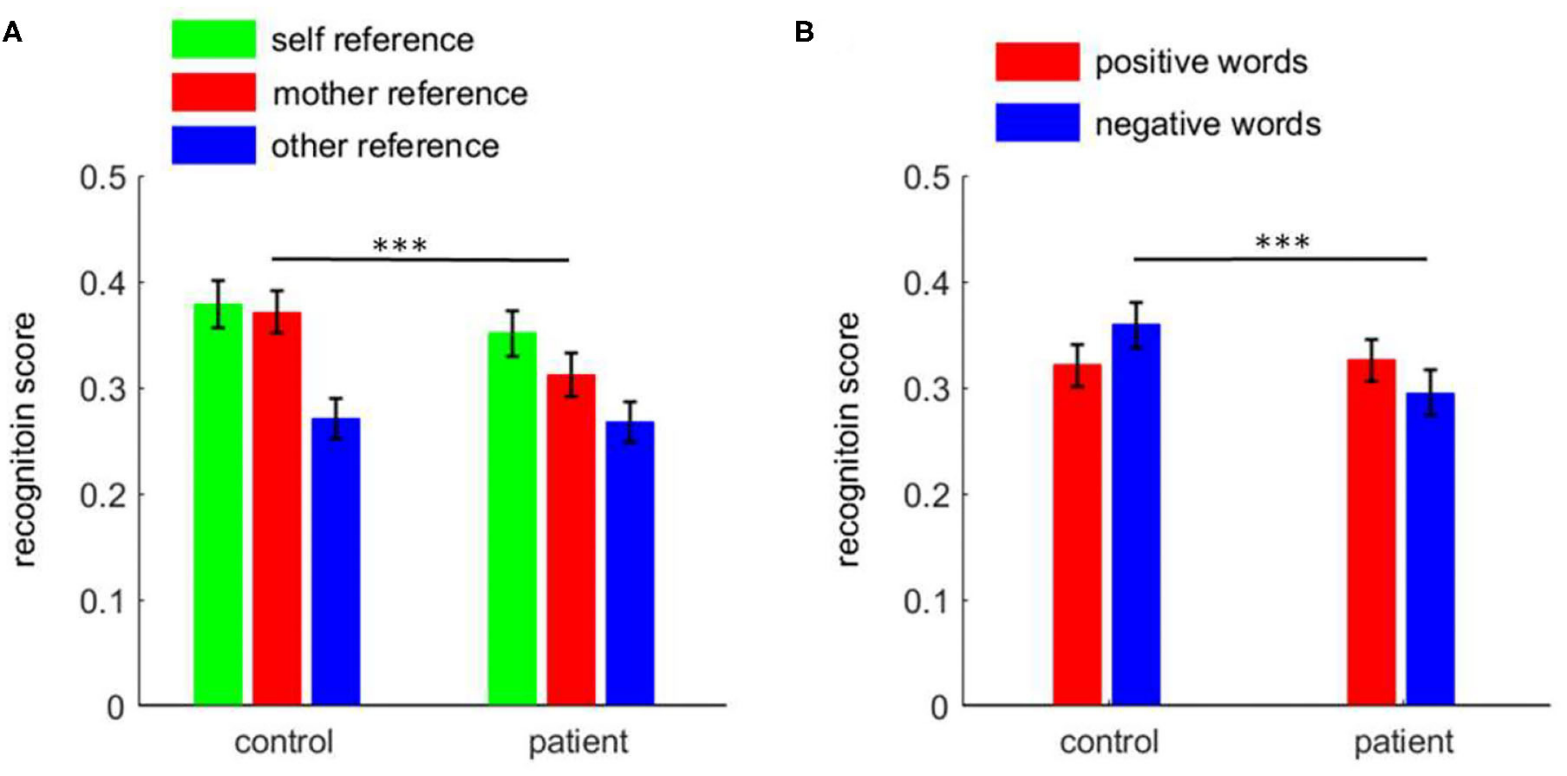
The recognition score among patients with first-episode schizophrenia and healthy control subjects. **(A)** the task effect; **(B)** the emotion effect. ***p < 0.001.

There was a two-way interaction between task and group [*F*_(2, 220)_ = 4.1, *p* = 0.018; Figure 2A)]. The simple effect analysis revealed that the recognition scores in patients revealed significant differences across the three conditions: self-reference (0.351 ± 0.022) > mother-reference (0.312 ± 0.020) > other-reference (0.268 ± 0.019; pairwise *p* ≤ 0.007). The recognition scores in the control group were different across conditions, i.e., self-reference (0.379 ± 0.022) and mother-reference conditions (0.371 ± 0.020; *p* = 0.999) were higher than the other-reference condition (0.271 ± 0.019; *p* < 0.001). While patients demonstrated a poorer recognition performance in the mother-referential condition than control participants (0.312 ± 0.020 vs. 0.371 ± 0.020, *p* = 0.041), the two groups did not show any significant difference in recognition scores in self- (*p* = 0.370) or other-referential conditions (*p* = 0.917).

Furthermore, there was an interaction between emotion and group [*F*_(1, 110)_ = 10.0, *p* = 0.002; Figure 2B]. While control subjects showed a significant negativity bias in the recognition phase (positive = 0.321 ± 0.020, negative = 0.359 ± 0.021, *p* = 0.016), patients, in contrast, had a significant positive bias (positive = 0.326 ± 0.020, negative = 0.295 ± 0.021, *p* = 0.045). Further analyses revealed that while patients showed poorer recognition performance for negative words compared to control participants (*p* = 0.029), the two groups did not show a significant difference in recognition scores for positive words (*p* = 0.884).

##### Bias Score

An independent samples *t*-test showed that the patient group (0.04 ± 0.08) had lower MRE bias scores than the control group (0.10 ± 0.12; *t* = 2.82, *p* = 0.006), while the two groups did not differ in the SRE bias scores (0.11 ± 0.11 vs. 0.08 ± 0.10, *t* = 1.26, *p* = 0.211).

##### Correlation Between Behavioral and Clinical Data

A significant correlation was found between the recognition score of negative self- and mother-reference words and the PANSS total score (self: *r* = −0.289, *p* = 0.031; mother: *r* = −0.271, *p* = 0.043). Moreover, the SRE bias scores were significantly correlated with the PANSS total score (*r* = −0.296, *p* = 0.027).

### ERP Results

#### Encoding Frontal P2

**Figure 3:**
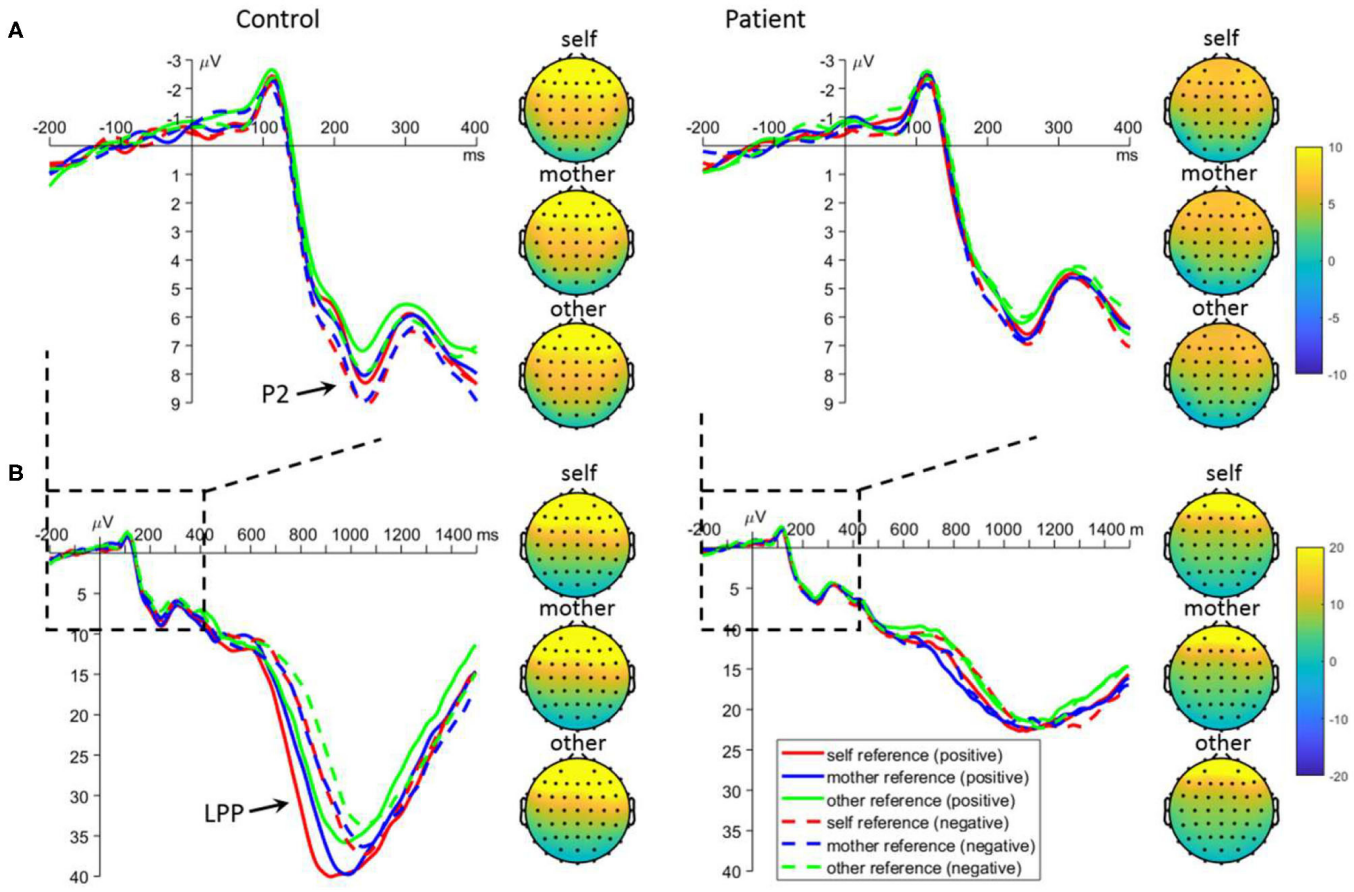
Mean event-related potential waveforms and topographies in response to negative and positive self-, mother-, and other-referential stimuli among patients with first-episode schizophrenia and healthy controls during encoding phase in frontal electrodes. Data were averaged across AF3, AF4, F3, and F4. The waveforms in subplot **(A)** are locally enlarged images of the waveforms in subplots **(B)** so as to highlight the P2 component.

For the mean amplitude of P2, the main effect of task was significant [*F*_(2, 220)_ = 3.4, *p* = 0.035]: the P2 was larger in the self- (7.0 ± 0.4 μV, *p* = 0.099) and mother-referential conditions (6.9 ± 0.4 μV, *p* = 0.088) than in the other-referential condition (6.3 ± 0.4 μV); no significant difference was observed between the former two conditions (*p* = 0.988). More importantly, the interaction between emotion and group was significant [*F*_(1, 110)_ = 5.3, *p* = 0.023; Figure 3]. Further analyses revealed that there was a significant emotion effect in the control group: the P2 was larger for negative (8.1 ± 0.5 μV) than positive referential words (7.3 ± 0.5 μV) in the control group (*p* = 0.005); however, the emotion effect was not observed in the patient group (positive = 5.8 ± 0.5 μV, negative = 5.7 ± 0.5 μV, *p* = 0.683). Additionally, the P2 amplitude was larger for negative-referential words in controls than in patients (*p* = 0.003), while the difference was marginally significant for positive-referential words (*p* = 0.064).

#### Encoding Frontal LPP

For the mean amplitude of LPP, the interaction between task and group was significant [*F*_(2, 220)_ = 4.8, *p* = 0.009; Figure 3]. Further analyses revealed that the LPP amplitudes were significantly influenced by task in the control group: the LPP was larger for self- (35.1 ± 2.4 μV; *p* < 0.001) and mother-referential words (33.6 ± 2.6 μV; *p* = 0.002) than for other-referential words (30.1 ± 2.3 μV), while no difference was found between self- and mother-referential conditions (*p* = 0.412). However, the LPP amplitudes were not influenced by task in the patient group (self = 21.5 ± 2.4 μV, mother = 22.7 ± 2.3 μV, other = 20.8 ± 2.4 μV; *p* ≥ 0.169). Furthermore, the LPP amplitudes were larger in the controls than in the patients across tasks (*p* < 0.010). Additionally, the interaction between emotion and group was significant [*F*_(1, 110)_ = 20.2, *p* < 0.001]. Further analyses revealed that there was a significant emotion effect in the control group: the LPP was larger for positive (35.0 ± 2.3 μV) than negative (31.1 ± 2.2 μV) referential words in the control group (*p* < 0.001); however, the emotion effect was not observed in the patient group (positive = 21.3 ± 2.4 μV; negative = 22.3 ± 2.2 μV, *p* = 0.144). Furthermore, the LPP amplitudes were larger for normal subjects than the patients for both positive and negative words (*p* ≤ 0.008).

For the peak latency of LPP, the main effect of emotion was significant [*F*_(1, 110)_ = 69.6, *p* < 0.001]: positive words evoked earlier LPP (1061.9 ± 12.0 ms) than negative words (1115.4 ± 12.5 ms). The main effect of group was significant [*F*_(1, 110)_ = 7.5, *p* = 0.007]: patients have slower LPP (1121.1 ± 16.7 ms) than the controls (1056.2 ± 16.7 ms).

#### Retrieval Parietal LPP

**Figure 4:**
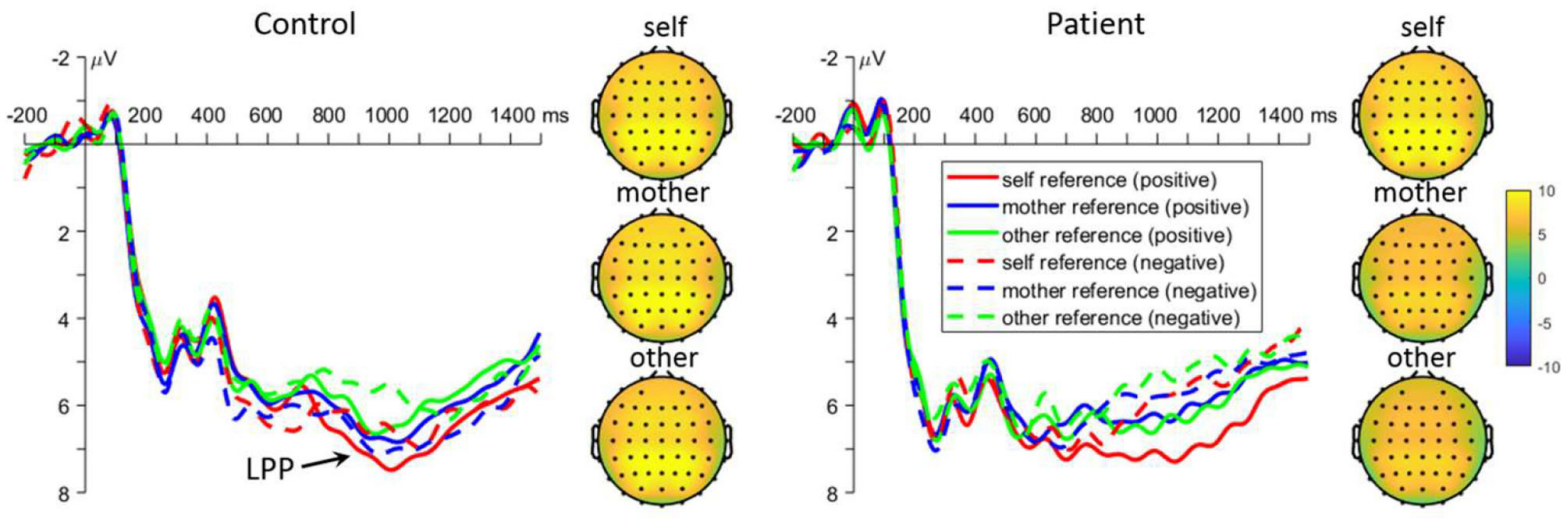
Mean event-related potential waveforms and topographies in response to negative and positive self-, mother-, and other-referential stimuli among patients with first-episode schizophrenia and healthy controls during retrieval phase in parietal electrodes. Data were averaged across at Pz, CPz, P1, and P2.

The interaction effect between task and group was significant [*F*_(2, 220)_ = 5.1, *p* = 0.009, Figure 4]. Further analyses revealed that self-reference words elicited larger LPP amplitudes than other-reference words in controls (6.2 ± 0.7 μV vs. 5.3 ± 0.7 μV, *p* = 0.024), while other-reference words elicited larger LPP amplitudes than mother-reference words in patients (7.0 ± 0.7 μV vs. 5.6 ± 0.7 μV, *p* = 0.013). There was no significant difference among all the other comparisons (all *p* > 0.050). Moreover, the interaction effect between emotion and group was significant [*F*_(1, 110)_ = 6.2, *p* = 0.014]. The simple effect analysis revealed that positive words evoked larger LPP amplitudes than negative words in the patients (7.2 ± 0.7 μV vs. 5.6 ± 0.6 μV, *p* < 0.001), while there were no significant differences in normal controls (5.8 ± 0.7 μV vs. 5.7 ± 0.6 μV, *p* = 0.707). Lastly, there was also significant three-way interaction effect [task by valance by group: *F*_(2, 220)_ = 3.4, *p* = 0.034]. Further analyses revealed that positive words evoked larger LPP amplitudes than negative words in patients under both self-and mother-reference conditions [self: 8.2 ± 0.8 μV vs. 5.1 ± 0.6 μV, *p* < 0.001; mother: 6.6 ± 0.7 μV vs. 4.7 ± 0.8 μV, *p* = 0.009], but not in the other-reference condition. Finally, positive self-related words elicited larger LPP amplitudes than both positive mother- and other-related words in patients (*p* = 0.015 and 0.014), and there was no significant difference between positive mother and other conditions (*p* = 0.998). In normal controls, positive self-related words elicited larger LPP amplitudes than other-related words (*p* = 0.019), but not than mother-related words (*p* = 0.357). For negative emotion, other-related words evoked larger amplitudes than mother- and self-related words in patients (*p* = 0.002 and 0.003), while no significant effect was found in normal subjects (all *p* > 0.050).

#### Correlation Between ERP and Clinical Data

The LPP evoked by both positive (*r* = −0.343, *p* = 0.010) and negative mother-reference words (*r* = −0.284, *p* = 0.034) in the retrieval phase were significantly correlated with the PANSS total scores.

### Correlation Between Behavioral and ERP Results

For patients, the LPP amplitude evoked by both positive self- and mother-reference words in the retrieval phase were positively correlated with the recognition score of positive self- and mother-reference words (self: *r* = 0.343, *p* = 0.010; mother: *r* = 0.293, *p* = 0.029). For controls, the P2 amplitude evoked by positive mother-reference words in the encoding phase was positively correlated with the recognition score for positive mother-judgment (*r* = 0.267, *p* = 0.046).

## Discussion

### Self- and Mother-Referential Processing in First-Episode Schizophrenia

Behaviorally, we found no significant difference in the SRE bias score and recognition score in self-referential conditions between the two groups, suggesting that first-episode patients have a relatively intact SRE. This finding is inconsistent with prior findings in non-first-episode patients with schizophrenia. The results of Zhao et al. (13) and Harvey et al. (19) indicated that the typical SRM bias is absent in schizophrenia patients; and they found that patients showed significantly reduced recognition sensitivity compared to controls for self-related information. Therefore, we suggest that self-referential processing or SRE dysfunction might not be a trait marker of schizophrenia as some researchers regarded (20, 21), and the impairment of self-referential processing may only present in the chronic course of the illness.

Meanwhile, this study did not find any significant difference between the recognition scores of self- and mother-related personality words in the control group, replicating the results of prior research (36, 41) and suggesting that the self includes individuals with whom one is intimate among the Chinese. Notably, the current study found that the recognition score of mother-related words was lower than that of self-judgment words in patients, and that their MRE bias score and recognition performance in mother-referential conditions were poorer than those of controls. This finding may be associated with the schizophrenic symptoms such as lack of emotion, reduced emotional interaction with intimate others, and increased suspicion of significant others. Prior fMRI research on chronic Western schizophrenia (42) found significant deficits when subjects were thinking about themselves, but the deficits were less evident when thinking about their mothers. Therefore, the current finding in the Chinese first-episode patients may imply that the mother-referential deficit is specific for first-episode schizophrenia; or alternatively, the difference between these two studies is due to cultural differences. We suggest future work to investigate this interesting topic.

The ERP results in healthy individuals demonstrated a typical task effect (self = mother > other) in both P2 and LPP amplitudes during encoding. In contrast, patients only exhibited the task effect during the P2 window. The results in controls are consistent with previous fMRI studies, demonstrating that self and mother representations are both associated with the mPFC activation (36). Results in patients demonstrated that the automatic semantic monitoring (reflected by P2) of self- and mother-information works relatively well, while mother-referential processing might be impaired during strategic cognitive processing (reflected by LPP). Moreover, patients showed smaller amplitudes of the P2 and LPP components across the three task conditions compared to healthy controls, similar with the previous findings observed in chronic schizophrenia patients (13). This result suggests that the frontal cortices of patients exhibit deficits in allocation of attention resources as well as strategic cognitive functions, regardless early or chronic schizophrenia.

In summary, the behavioral results confirmed the hypothesis that core self-function is preserved but relational-self is changed in first-episode schizophrenia. EEG results further provide electrophysiological evidence for this finding.

### Absence of Negative Memory Bias in the First-Episode Schizophrenia

Consistent with our hypothesis, control subjects showed a significant negativity bias in the recognition scores. Patients, in contrast, had a significant positive bias. Additionally, patients showed a poorer recognition performance for negative words compared to control participants. The results of controls were consistent with prior research (22, 24) and consistent with the well-known effect of enhanced memory for negative material, i.e., remembering negative stimuli is more critical than remembering positive stimuli for human survival (43). However, a negative memory bias with an adaptive value was absent in the first episode-schizophrenia. At the ERP level, a negative bias was observed in controls during the automatic processing stage (demonstrated by greater P2 amplitudes for negative relative to positive items) while it was not observed in the patients at the encoding phase. However, during the retrieval stage, patients showed a positive bias (positive words evoked larger LPP amplitudes than negative words under both self- and mother-reference conditions). This finding provides evidence for the hypothesis that the abnormality of self-referential processing in first-episode schizophrenia occurs not only in the encoding stage but also in the retrieval stage. In our opinion, the absence of a negative memory bias in the first episode schizophrenia might be due to the dysfunction of automatic attention preference to negative information, or the inhibition for the extraction of negative information as to maintain self-esteem. In line with this idea, there was positive correlation between the LPP amplitude and the recognition score in patients.

Moreover, patients made more negative assessments about themselves as they endorsed more unpleasant words as self-referential compared with normal controls. In addition, relative to normal subjects, schizophrenic patients were slower with positive words but faster with negative words in self-relevant conditions. Prior research in chronic schizophrenia outpatients found that compared to controls, patients were less likely and slower to endorse positive self-attributes, and more likely and quicker to endorse negative self-attributes (42). These results showed that both the first episode and chronic schizophrenia patients have a negative self-identity. Compared to healthy controls, schizophrenic patients were more likely to endorse negative mother-attributes and they were slower to endorse mother-related words, especially for negative mother-related personality traits words. Consistent with this finding, studies on individuals with high psychosis proneness found that the subjects attributed fewer positive traits to acquaintance others than subjects with low psychosis proneness (8). Dysfunctional strategies for avoiding low self-esteem underlie the positive psychotic symptoms such as paranoia-inducing explanations (30, 44). Therefore, patients may display distorted attributions of less positive or more negative traits to others. It is worth noting that both normal controls and the patients responded slowest to endorse mother-related negative words compared to the other conditions, which may be due to their sensitive and suspicious symptoms toward significant others.

### Clinical Implications

The current study shows that the impairment of self-referential processing occurs in the early stage of the disease and the characteristics of the dysfunction are different from those of chronic schizophrenia. While the self-impairment in first-episode schizophrenia is manifested in the relational self, the core self-dysfunction is found in the chronic schizophrenia (9–11, 13). These findings indicate the target for the treatment in different disease stages. Specifically, first-episode schizophrenic patients have negative identification with themselves and the significant others. Meanwhile, they have insufficient attention and inhibition of extraction of negative information. All these may be related to their use of abnormal psychological strategies in order to self-defense (23) or avoid low self-esteem (30, 44). Meanwhile, correlations were observed between the PANSS total score and the SRE bias score, the recognition score of negative self- and mother-reference words, and the retrieval LPP evoked by mother-reference words, indicating that the severity of the disease has an effect on the self-reference processing preference, especially on negative interdependent self-reference processing. It suggests that the relational-self impairment in first-episode schizophrenia may recover with the improvement of symptoms with the use of drugs.

### Limitations and Future Work

The limitation of this study, which is also a problem that needs general attention in the study of self-referential processing, especially when examining the emotional effects, is that the valence and arousal of the positive and negative words were not balanced, although we do not view our results as reflective of this, as the ERP results show different patterns in different ERP components and different processing stages in both controls and patients. In addition, the age range of the participants is large which might be a potential confounding factor in this study, since the relationship to one's mother may undergo some changes at different ages. Furthermore, the N400 is an important and interesting component in the study of self-referential processing in schizophrenia. Previous studies found no N400 effect in non-first episode schizophrenia, which may be associated with a disordered self-concept (4). However, our experimental design did not include enough trial numbers for N400 analysis (see trial numbers per condition in supplementary material). Based on the behavioral results of this study, we speculated that the N400 effect of patients with first-episode schizophrenia would be intact during self-referential processing, but the N400 effect might be diminished during mother-referential processing. We therefore suggest testing this hypothesis in future works.

## Conclusions

In conclusion, the present study investigated self-referential processing in first-episode schizophrenia using both behavioral and ERP techniques. It was found that although first-episode schizophrenic patients demonstrated a typical SRE, like healthy controls, their interdependent self-representation is changed as their self does not include the intimate other (i.e., mother). The impaired preference of mother-referential processing mainly emerged during the strategic cognitive processing of mother-related information at the encoding phase. Moreover, patients have negative assessments with regard to themselves and the intimate others, and the negative memory bias exhibited in controls was abolished in patients, which was not only associated with the disappearance of the attention bias for negative information, but also related to dysfunction in the retrieval of negative information. These results deepen our understanding of the behavioral features and neural correlates of impaired reflective self-function in first-episode schizophrenia.

## Data Availability Statement

The raw data supporting the conclusions of this article will be made available by the authors, without undue reservation.

## Ethics Statement

The studies involving human participants were reviewed and approved by The Ethics Review Board at the Beijing Huilongguan Hospital. The patients/participants provided their written informed consent to participate in this study.

## Author Contributions

YanZ wrote the first draft of the manuscript. ZW made critical revisions. YueZ, YuaZ, JZ, and DL collected the behavioral and ERP data. CX collected the clinical data. ST supervised this research and revised the manuscript. DZ analyzed data and revised the manuscript. All authors contributed to the article and approved the submitted version.

## Conflict of Interest

The authors declare that the research was conducted in the absence of any commercial or financial relationships that could be construed as a potential conflict of interest.
